# Increased MANF Expression in the Inferior Temporal Gyrus in Patients With Alzheimer Disease

**DOI:** 10.3389/fnagi.2021.639318

**Published:** 2021-04-29

**Authors:** Xue-Chun Liu, Xiu-Hong Qi, Hui Fang, Ke-Qing Zhou, Qing-Song Wang, Gui-Hai Chen

**Affiliations:** ^1^Department of Neurology (Sleep Disorders), The Affiliated Chaohu Hospital of Anhui Medical University, Hefei, China; ^2^Department of Neurology, Chinese PLA Clinical College, Anhui Medical University, Hefei, China; ^3^Hefei National Laboratory for Physical Sciences at the Microscale, School of Life Sciences, Chinese Academy of Sciences Key Laboratory of Brain Function and Diseases, University of Science and Technology of China, Hefei, China

**Keywords:** Alzheimer disease, cerebral cortex, endoplasmic reticulum stress, MANF, hyperphosphorylated tau, senile plaque

## Abstract

Alzheimer disease (AD) is an aging-related disorder linked to endoplasmic reticulum (ER) stress. The main pathologic feature of AD is the presence of extracellular senile plaques and intraneuronal neurofibrillary tangles (NFTs) in the brain. In neurodegenerative diseases, the unfolded protein response (UPR) induced by ER stress ensures cell survival. Mesencephalic astrocyte-derived neurotrophic factor (MANF) protects against ER stress and has been implicated in the pathogenesis of AD. MANF is expressed in neurons of the brain and spinal cord. However, there have been no investigations on MANF expression in the brain of AD patients. This was addressed in the present study by immunohistochemistry, western blotting, and quantitative analyses of postmortem brain specimens. We examined the localization and expression levels of MANF in the inferior temporal gyrus of the cortex (ITGC) in AD patients (*n* = 5), preclinical (pre-)AD patients (*n* = 5), and age-matched non-dementia controls (*n* = 5) by double immunofluorescence labeling with antibodies against the neuron-specific nuclear protein neuronal nuclei (NeuN), ER chaperone protein 78-kDa glucose-regulated protein (GRP78), and MANF. The results showed that MANF was mainly expressed in neurons of the ITGC in all 3 groups; However, the number of MANF-positive neurons was significantly higher in pre-AD (Braak stage III/IV) and AD (Braak stage V/VI) patients than that in the control group. Thus, MANF is overexpressed in AD and pre-AD, suggesting that it can serve as a diagnostic marker for early stage disease.

## Introduction

Alzheimer disease (AD) is a progressive neurodegenerative disease with insidious onset (Mathys et al., 2019) that constitutes a major public health burden (Scheltens et al., 2016; Alzheimer’s Association, 2020). The prevalence of AD among people over the age of 65 years is estimated to be 10–30%, which is increasing with the aging of the global population (Masters et al., 2015). AD is characterized by a progressive decline in cognitive function and neuronal loss (Pietronigro et al., 2016). There are two types of brain lesion that are the pathologic hallmarks of AD: extracellular senile plaques composed of amyloid β-peptide (Aβ), and intraneuronal neurofibrillary tangles (NFTs) consisting of paired helical filaments of hyperphosphorylated tau protein (Braak and Del Tredici, 2018). AD patients exhibit different degrees of nucleolar pyknosis in neurons or even the disappearance of neurons. The pathogenesis of AD has been linked to dysfunction of the endoplasmic reticulum (ER) (Mukherjee and Soto, 2011; Cabral-Miranda and Hetz, 2018), which is the site of protein folding and secretion in eukaryotic cells (Gerakis and Hetz, 2018). ER stress, which is induced by the accumulation of unfolded or misfolded proteins in the ER (Ghemrawi and Khair, 2020), has been proposed as a mechanism underlying Aβ-induced Alzheimer-like neuropathology (Goswami et al., 2020). Although homeostatic mechanisms such as the unfolded protein response (UPR) can restore normal ER function (Rahman et al., 2018), prolonged ER stress can lead to cell dysfunction and death (Bravo et al., 2013; Lu et al., 2014).

Mesencephalic astrocyte-derived neurotrophic factor (MANF) – originally named arginine-rich protein (ARP) or arginine-rich mutated in early tumors (ARMET) – is an evolutionarily conserved secreted protein expressed in the rodent brain that has been shown to play a protective role in ER stress (Apostolou et al., 2008; Wang et al., 2014). Like cerebral dopamine neurotrophic factor (CDNF), MANF is classified as a neurotrophic factor (Lindholm and Saarma, 2010; Lindahl et al., 2017) that participates in the UPR (Apostolou et al., 2008). MANF was shown to rescue neurons from apoptosis and ER stress (Hellman et al., 2011), and knocking down MANF expression induced the UPR and increased the neurotoxic effects of Aβ (Xu et al., 2019). Additionally, chronic activation of the UPR in the brain has been reported in MANF-deficient mice (Pakarinen et al., 2020). Glucose-regulated protein (GRP)78 is an ER stress-associated marker (Sakono and Kidani, 2017) and UPR-regulated chaperone that interacts with MANF (Yan et al., 2019). In general, MANF is upregulated and plays a protective role in the response to ER stress (Xu et al., 2019). MANF exerted neuroprotective effects against ethanol-induced neurodegeneration by alleviating ER stress, which may be relevant to other ER stress-related neurodegenerative diseases (Wang Y. et al., 2021).

MANF has shown protective effects in animal models of AD (Xu et al., 2019), Parkinson disease (Voutilainen et al., 2009), spinocerebellar ataxia (Yang et al., 2014), ischemic brain damage (Airavaara et al., 2010), retinal degeneration (Lu et al., 2018), cardiac ischemia (Arrieta et al., 2020), and liver injury (Sousa-Victor et al., 2019). Given the diverse pathologies that MANF can alleviate, its involvement in diseases related to the activation of the UPR is expected. However, to date there have been no reports on the expression of MANF in the brain of AD patients.

The inferior temporal gyrus of the cortex (ITGC) is a key brain area involved in cognitive functions including memory, auditory cognition, and semantics (Meunier and Barbeau, 2013). The ITGC plays an important role in verbal fluency, which is affected soon after the onset of AD (Scheff et al., 2011). The hippocampus, a region severely affected in AD, is connected to the ITGC (Mégevand et al., 2017). To address the above point, the present study examined the subcellular localization and expression of MANF in the ITGC of human brain specimens from pre-AD and AD patients in order to clarify its role in AD pathogenesis.

## Materials and Methods

### Brain Specimens

Paraffin-embedded postmortem human brain tissue specimens were provided by the Netherlands Brain Bank (NBB) (Amsterdam, Netherlands). The brains were donated to research after patients or their closest relatives provided written, informed consent. AD pathology was evaluated according to Braak stage (Braak and Braak, 1991). Ten AD cases (age range, 59–95 years; male-to-female ratio, 2:3) and 5 sex- and age-matched control cases (age range, 60–93 years; male-to-female ratio, 2:3) were included in the analysis. The AD cases were further classified into pre-AD (Braak stages: III/IV) and AD (Braak stages: V/VI) (*n* = 5 each). Non-dementia control cases (Braak stage: 0/I) had no known clinical history of dementia, and the cause of death was unrelated to the central nervous system. Demographic and clinicopathologic data for the samples are shown in Table 1.

**TABLE 1:** Demographic and clinicopathologic information of the samples.

NBB no.	Autopsy	Braak stage		Group	Age (years)	Sex	PMD (h)	BW (g)	CSF pH
		
		NFT	Amyloid						
15033	S15/033	0	A	CON	93	M	07:40	1155	6.20
14043	S14/043	0	O	CON	60	F	08:10	1310	6.58
01045	S01/115	I	B	CON	83	M	04:35	1367	6.49
02018	S02/043	I	B	CON	92	F	07:00	1193	6.45
04026	S04/074	I	B	CON	91	F	07:45	1054	6.90
14014	S14/014	III	B	Pre-AD	90	F	06:05	1255	6.12
14020	S14/020	III	O	Pre-AD	92	F	06:35	1305	6.12
95059	S95/140	IV	NA	Pre-AD	86	F	03:20	995	7.14
08075	S08/241	IV	C	Pre-AD	88	M	05:00	1296	6.45
09096	S09/301	IV	C	Pre-AD	92	M	08:25	1117	6.14
97015	S97/045	V	NA	AD	85	F	03:10	1044	6.90
00054	S00/115	V	C	AD	59	M	07:45	1171	6.29
00119	S00/264	V	C	AD	85	F	06:10	1003	6.65
11121	S11/121	V	NA	AD	95	M	07:00	1143	6.18
02069	S02/203	VI	C	AD	70	F	08:15	876	6.30

*AD, Alzheimer disease; BW, brain weight; CON, non-dementia control; CSF, cerebrospinal fluid; F, female; M, male; NA, not available; NBB, Netherlands Brain Bank; NFT, neurofibrillary tangles; PMD, postmortem delay; pre-AD, preclinical Alzheimer disease.*

### Tissue Preparation

Paraffin blocks containing the ITGC were stored at room temperature under protection from light. Serial coronal sections were cut on a microtome (RM2235; Leica, Wetzlar, Germany) at a thickness of 6 μm and stored at room temperature. We selected 1 section every 150 μm (or approximately every 25 sections) for a total of 45 sections from the ITGC, as well as 1 section each from the anterior, middle, and posterior cortices. Thus, 45 brain tissue sections were used for MANF immunohistochemistry from pre-AD, AD, and non-dementia control specimens for quantification of MANF-immunoreactive neurons in the ITGC. Additionally, in order to detect pathologic lesions (Aβ and tau) and determine whether MANF is primarily expressed in neurons and determine its subcellular localization, several adjacent sections were randomly selected from pre-AD, AD, and non-dementia control.

### Specific Assessment of MANF Antibodies

To ensure that the rabbit polyclonal anti-MANF antibody used in this study (ARMET/ARP; cat. no. Ab67271; Abcam, Cambridge, United Kingdom) could detect MANF in human brain specimens, we tested its specificity in cell lysates of HEK 293T and SHSY5Y cell line and homogenates of human brain temporal cortex tissue by western blotting under denaturing conditions as previously described (Huang et al., 2020). The SHSY5Y (Catalog number TCHu97), HEK293T (Catalog number GNHu17) cells were purchased from the typical culture preservation committee of Chinese academy of sciences. To further confirm the specificity of the anti-MANF antibody, the levels of MANF expression in hepatocellular tissues of hepatocyte-specific MANF-knockout (HKO) control mice and wild-type (WT) control mice were measured by immunohistochemistry. In previous studies, the efficiency of MANF knockout from the HKO control mice have been detected with the use of western blotting (Yang et al., 2021). The hepatocellular tissue sections of the HKO control mice and WT control mice were provided by Prof. Yuxian Shen of the Anhui Medical University.

### Immunohistochemistry

Brain sections were deparaffinized to water according to standard procedures. After washing with phosphate-buffered saline (PBS; pH value 7.2–7.4; 3 × 10 min), antigen retrieval was performed by boiling the sections in citric acid buffer and steaming for 1 min 15 s. The sections were left to cool at room temperature for about 30 min, then flushed with warm water (50°C) for 5 min and washed with PBS (3 × 10 min). Endogenous peroxidase activity was quenched by incubating the sections in a solution of 3% H_2_O_2_ and methanol for 10 min at 25°C. The tissue was blocked in 10% sheep serum albumin solution at 37°C for 1 h, followed by overnight incubation at 4°C with rabbit polyclonal anti-MANF antibody at 1:400 dilution. The following day, the sections were warmed at room temperature for 15 min in a covered humid chamber, then washed in PBS (3 × 10 min) before incubation with biotin-conjugated goat anti-rabbit IgG for 15 min at room temperature (≥25°C). After washing in PBS (3 × 10 min), diaminobenzidine reagent was added dropwise, and the colorimetric reaction was allowed to proceed for 1–1.5 min. The sections were washed and then stained with hematoxylin, dehydrated through a graded series of alcohol and xylene, and mounted with neutral gum for observation under a light microscope (CX43; Olympus, Tokyo, Japan). Immunohistochemical detection of tau and Aβ was performed using monoclonal antibodies against phosphorylated (p-)tau (Ser202, Thr205) (AT8; Thermo Fisher Scientific, Waltham, MA, United States; cat. no. MN1020) and Aβ_17–24_ (4G8; Biolegend, San Diego, CA, United States; cat. no. 800701), both used at 1:200 dilution. Immunohistochemistry was performed on liver sections as previous described (Wang P. et al., 2021).

To determine whether MANF is mainly expressed in neurons and to determine its subcellular localization, we performed double immunofluorescence labeling of MANF/neuronal nuclei (NeuN) and MANF/GRP78 as previously described (Shen et al., 2012; Gao et al., 2018; Herranen et al., 2020), with minor modifications to the protocol. The following antibodies were used: rabbit polyclonal anti-MANF (1:400 dilution); mouse monoclonal anti-NeuN (clone A60, cat. no. MAB377; Abcam) (1:400 dilution); and rabbit polyclonal anti-GRP78 (GRP78 BiP, cat. no. ab21685; Abcam) (1:200 dilution). Nuclei were stained with 4’,6-diamidino-2-phenylindole (cat. no. C1005; Beyotime, Shanghai, China). The specificity of these antibodies has been reported in previous studies (Zhu et al., 2007; Shen et al., 2012; Braak and Del Tredici, 2018; Adaikkan et al., 2019; Xu et al., 2019).

### Measurements

Images of the brain sections were obtained with a digital slide scanner (Pannoramic MIDI; 3DHISTECH, Budapest, Hungary) and Case Viewer software (3DHISTECH). Quantitative analysis of the density of neurons expressing MANF in the cytoplasm was carried out using ImageJ software (Schneider et al., 2012). The diameters of the region of interests (ROIs) in nucleoli were measured with MetaMorph software (Molecular Devices, San Jose, CA, United States). The methods used for region selection and measurement have been described elsewhere (Hu et al., 2002, 2003; Thangavel et al., 2008). Briefly, the cortical selection in each tissue section was defined in the 5 ROIs (size: 600 × 317 μm) on the digitized autoradiograms using Case Viewer software, and the area was measured. Each selection was drawn from the cortical surface extending perpendicularly to the gray and white matter boundary. Up to 5 selections were defined for each ROI but in some ROIs, the number of selections was limited by loss of tissue integrity. In each image of the ITGC acquired under high magnification (40× objective), the average optical density of immunopositive areas was calculated for each visual field; the density of neurons expressing MANF in the cytoplasm was determined. Additionally, the diameter of nucleoli of nucleolated neuronal profiles from 5 ROIs per ITGC tissue section under high magnification (40× objective) was measured by an investigator who was blinded to the clinicopathologic data of the subjects.

### Statistical Analysis

Statistical analyses were performed using SPSS v22.0 (SPSS Inc., Chicago, IL, United States). Data are expressed as mean ± standard deviation. Based on Shapiro–Wilk test, almost all data were skewed, so non-parametric tests were used. Differences between groups were evaluated with the Kruskal–Wallis *H* test for multiple comparisons. Two-way analysis of variance was used to compare MANF expression in the ITGC among pre-AD, AD, and non-dementia control cases. *P* < 0.05 was considered significant.

## Results

### Clinicopathologic Information of pre-AD, AD, and Control Cases

**FIGURE 1:**
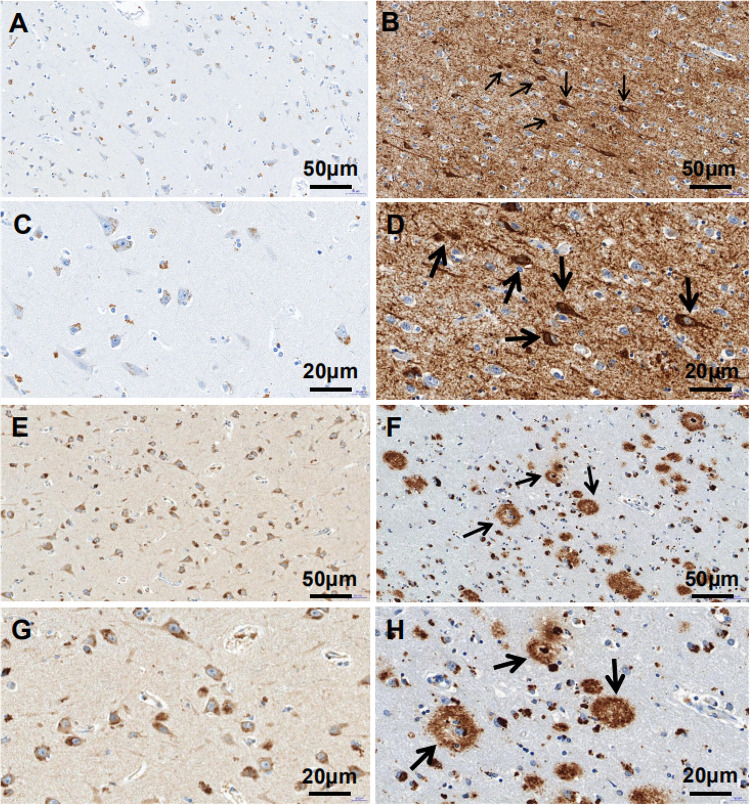
p-tau and Aβ_17–24_ expressions in the ITGC detected by immunohistochemistry. **(A–H)** p-tau and Aβ_17–24_ immunoreactivity in control subjects (left) and AD patients (right). Patients with AD showed extensive accumulation of Aβ plaques [arrowheads in panels **(F,H)**] and NFTs [arrowheads in panels **(B,D)**] in the ITGC. Scale bar, 50 μm **(A,B,E,F)**, 20 μm **(C,D,G,H)**.

The clinicopathologic information for the study population is summarized in Table 1. There were no differences in age (*P* = 0.428), postmortem delay (*P* = 0.650), body weight (*P* = 0.125), and cerebrospinal fluid pH (*P* = 0.404) among pre-AD and AD patients and control subjects. Immunohistochemical analysis revealed that p-tau immunoreactivity was predominantly in the ITGC and was higher in AD patients (NBB no. 00119; Autopsy S00/264) compared to control subjects (NBB no. 01045; Autopsy S01/115). Aβ_17–24_ (4G8) expression was also higher in AD cases (NBB no. 11121; Autopsy S11/121) than in controls (NBB no. 15033; Autopsy S15/033) (Figure 1).

### Specificity of the Anti-MANF Antibody

**FIGURE 2:**
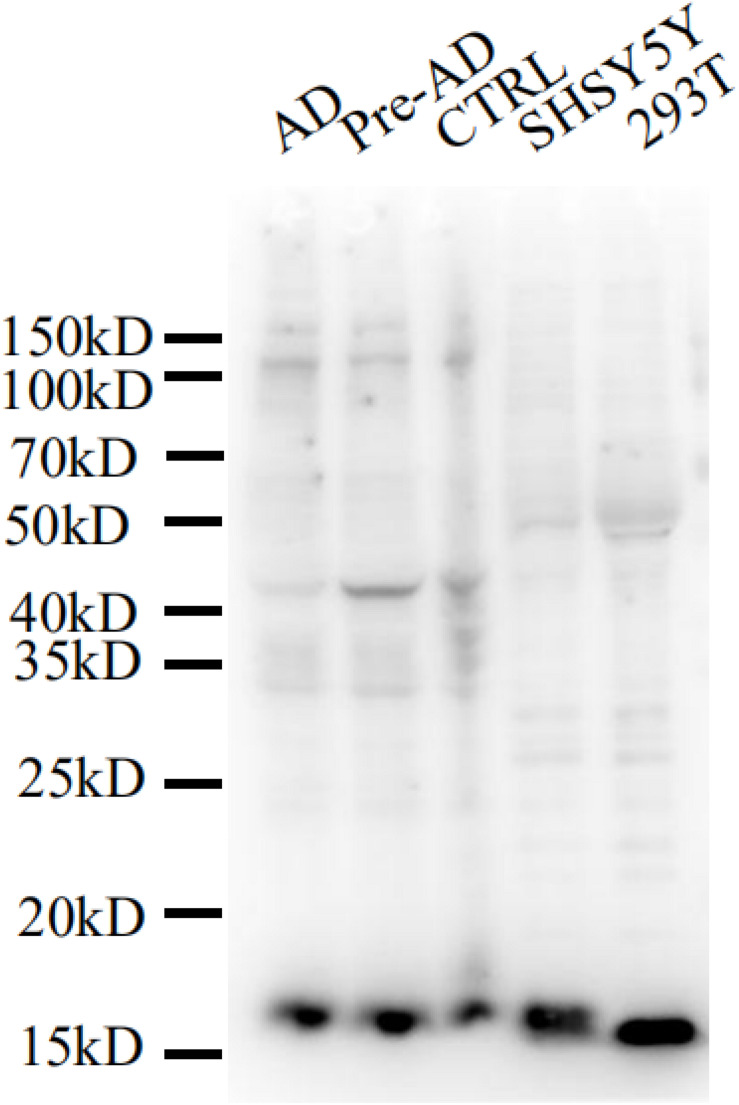
Mesencephalic astrocyte-derived neurotrophic factor levels detected by western blotting with a polyclonal antibody. Cell lysis solutions and human brain temporal cortex protein extracts were probed with a polyclonal anti-MANF antibody to evaluate specificity. The immunoblot shows a clear single band of ∼18 kDa in all protein extracts and cell lysis solutions.

**FIGURE 3:**
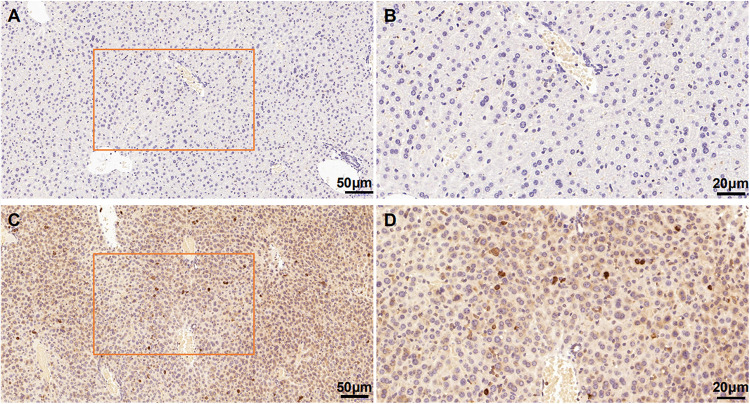
Mesencephalic astrocyte-derived neurotrophic factor levels in the HKO control mice and WT control mice detected by immunohistochemistry with polyclonal antibodies. No positive staining was detected in the hepatocellular tissues of the HKO control mice **(A,B)**. MANF was detected in the hepatocellular tissues of the WT control mice **(C,D)**. Scale bar: 50 μm **(A,C)** and 20 μm **(B,D)**.

We evaluated the specificity of the polyclonal anti-MANF antibody by western blotting using cell cultures and human brain temporal cortex extracts. The antibody recognized a single band at ∼18 kDa – which is the known molecular weight of MANF protein (Figure 2). Furthermore, we have not detected MANF expression in hepatocellular tissues of HKO control mice by immunohistochemical staining, compared with WT control mice (Figure 3).

### Nucleoli of ITGC Neurons Are Reduced in Size in Pre-AD and AD

The diameter of neuronal nucleoli of ITGC neurons was significantly smaller in pre-AD and AD patients than in control subjects (*P* < 0.05; Table 2).

**TABLE 2:** Mesencephalic astrocyte-derived neurotrophic factor expression in the inferior temporal gyrus cortex in pre-AD and AD patients and non-dementia controls.

Variable	AD	Pre-AD	Control
Diameter of nucleoli (μm)	3.68 ± 0.77**	3.70 ± 0.82**	4.16 ± 1.22
Cytoplasmic MANF-positive neurons/1 mm^2^	115.81 ± 21.24**	116.58 ± 21.99**	100.85 ± 19.91

Values represent mean ± standard deviation; **P < 0.05 vs. control group (two-way analysis of variance). AD, Alzheimer disease; MANF, mesencephalic astrocyte-derived neurotrophic factor; pre-AD, preclinical Alzheimer disease.

### Distribution of MANF-Positive Neurons in the ITGC and Subcellular Localization of MANF

**FIGURE 4:**
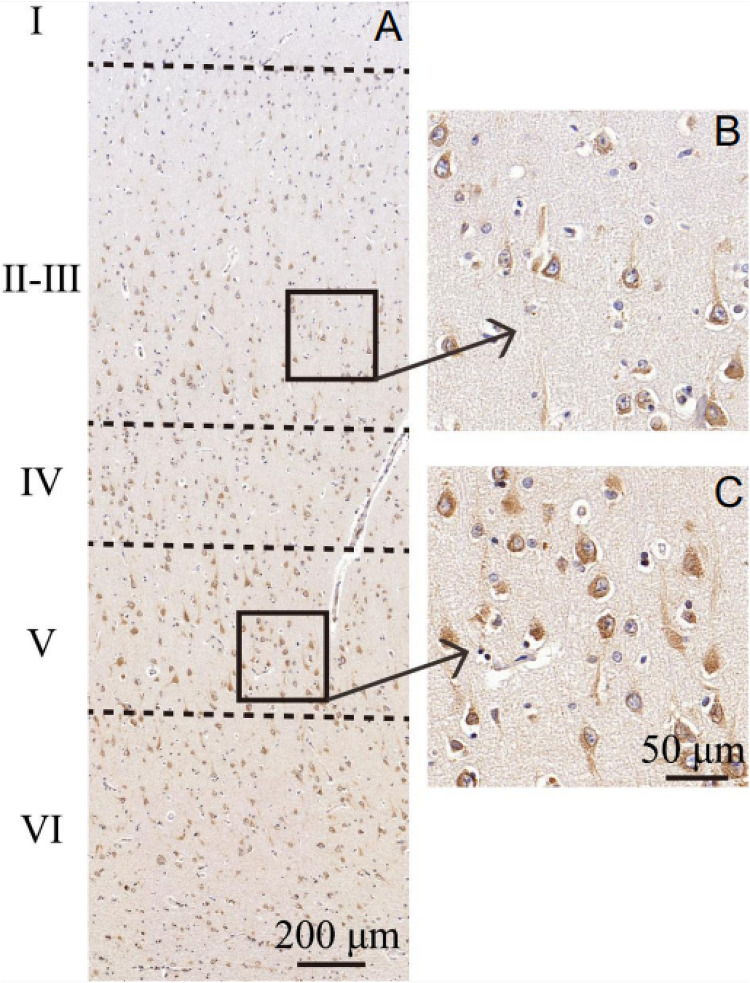
Cytoarchitecture of the ITGC. **(A–C)** Organization of cortical laminae visualized in tissue sections immunolabeled with an antibody against MANF. Layers are indicated by roman numerals. Scale bar, 200 μm **(A)** and 50 μm **(B,C)**.

**FIGURE 5:**
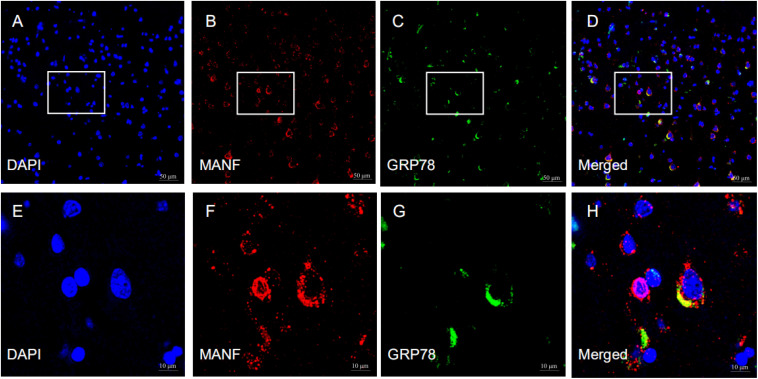
Representative images of the expression of MANF and GRP78 in the ITGC. **(A–H)** The pictures above are representative images of immunofluorescence labeling for MANF (red) and GRP78 (green). The nuclei were stained with DAPI (blue). Picture D is a merged image of panels **(A–C)**. The magnified images show the expression and cellular distribution of MANF and GRP78, respectively. Scale bar: 50 μm **(A–D)** and 10 μm **(E–H)**.

**FIGURE 6:**
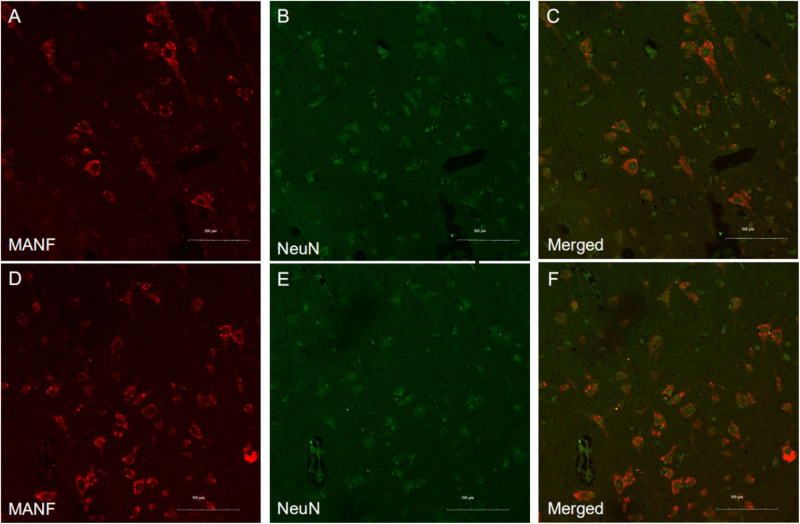
Representative images of MANF and NeuN expression in the ITGC. **(A–F)** Representative micrographs of double immunolabeling with antibodies against MANF (red) and NeuN (green). MANF was mainly detected in neurons. Scale bar: 100 μm **(A–F)**.

MANF expression was detected in the ITGC of human brain specimens (NBB no. 02018; Autopsy S02/043). Neurons in layer IV and V had especially strong MANF immunoreactivity (Figure 4). Double immunofluorescence labeling showed partial superimposition of GRP78 and MANF, which were mainly distributed throughout the ER (NBB no. 97015; Autopsy S97/045) (Figure 5). A large number of neurons in the ITGC express both MANF and NeuN, indicating that MANF is a protein expressed primarily in neurons (NBB no. 00119; Autopsy S00/264) (Figure 6).

### Mesencephalic Astrocyte-Derived Neurotrophic Factor Is Overexpressed in the ITGC in Pre-AD and AD

**FIGURE 7:**
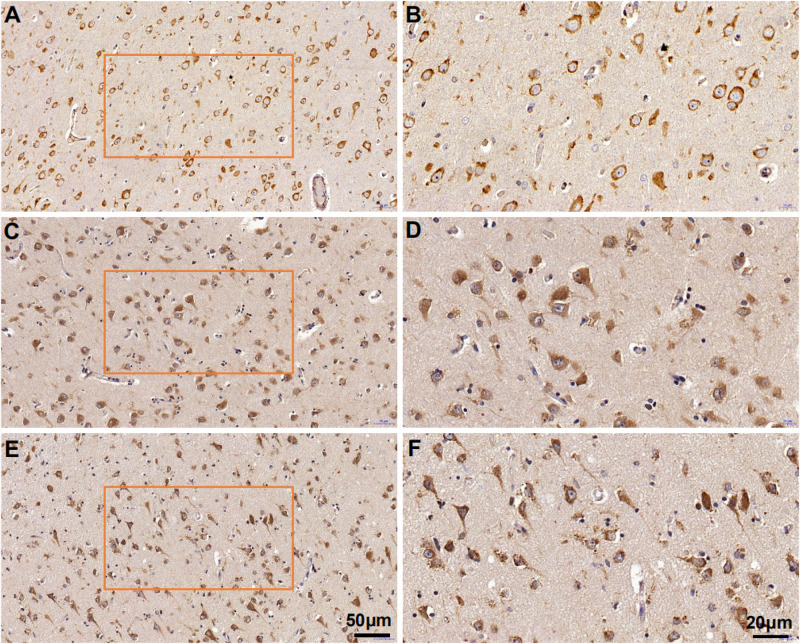
Mesencephalic astrocyte-derived neurotrophic factor expression in the ITGC of AD, pre-AD, and control cases detected by immunohistochemistry. **(A–F)** MANF expression in non-dementia control **(A,B)**, pre-AD **(C,D)**, and AD **(E,F)** samples shown at low (20×) **(A,C,E)** and high (40×) **(B,D,F)** magnification.

**FIGURE 8:**
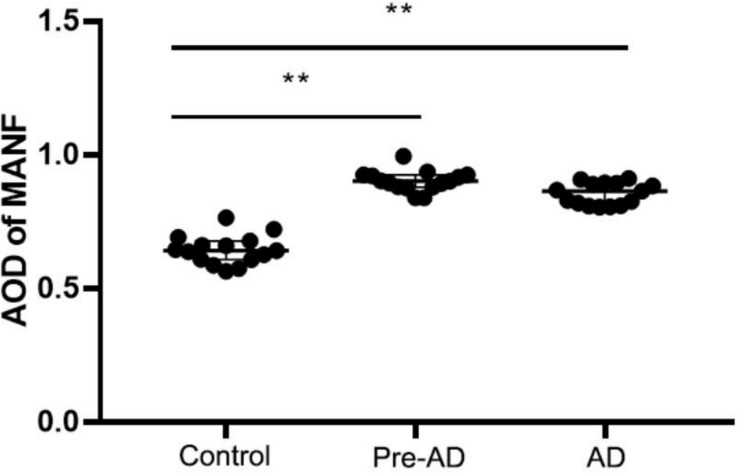
Increased MANF expression in the ITGC of pre-AD and AD groups. Overall expression in both groups was higher than in the control group, while the expression level was lower in the AD group than in the pre-AD group, although the difference was non-significant. **P < 0.05 vs. control group.

Mesencephalic astrocyte-derived neurotrophic factor expression in the ITGC of pre-AD, AD, and non-dementia control cases was evaluated by immunohistochemistry. MANF immunoreactivity was observed in all three groups, mainly in neurons (Figure 7). The rank order of expression level was pre-AD > AD >> control (Figure 8). Additionally, the number of neurons in the ITGC with cytoplasmic MANF expression per unit area (1 mm^2^) was higher in pre-AD and AD patients than non-dementia control cases. MANF was mainly expressed in the cytoplasm of neurons (Table 2).

## Discussion

The results of this study demonstrate that proteins associated with AD pathogenesis were more highly expressed in the ITGC of patients with AD than in control subjects, which is consistent with previous reports (Zhu et al., 2007; Lacosta et al., 2017). These AD biomarkers include p-tau and Aβ_17–24_, which are components of NFTs and amyloid plaques, respectively.

Mesencephalic astrocyte-derived neurotrophic factor is an evolutionarily conserved protein with both cytoprotective and immunomodulatory effects (Neves et al., 2016) that is highly expressed in the developing mammalian cortex and is involved in neurite extension and the regulation of ER homeostasis in neurons (Adaikkan et al., 2019). MANF was shown to play a protective role in cell survival by attenuating the neurotoxicity resulting from ER stress (Xu et al., 2019). Moreover, treatment with recombinant MANF or MANF overexpression alleviated Aβ-induced UPR activation caused by ER stress, while knocking down MANF promoted UPR activation and enhanced the toxicity of Aβ (Xu et al., 2019). Exogenous MANF stimulated nerve repair in dopaminergic neurons (Petrova et al., 2003; Voutilainen et al., 2009; Hao et al., 2017; Liu et al., 2018) and although it was not essential for neuron survival in mouse embryo, the endogenous protein was shown to be necessary for maintaining neuronal ER homeostasis both *in vitro* and *in vivo* (Pakarinen et al., 2020). As an ER stress-associated protein, MANF has been implicated in chronic stress and multiple neurodegenerative diseases including AD (Zhu et al., 2017).

In our study, we examined the expression of MANF in the ITGC of human brain specimens from pre-AD and AD patients and non-dementia control cases by immunohistochemistry. A representative Western blot result shows that the MANF protein was stably expressed in HEK 293T and SHSY5Y cells. However, compared with the WT control mice, MANF did not express in the hepatic cells of the HKO control mice. The reported specificity of the anti-MANF antibody (Lindholm et al., 2008; Yang et al., 2017) was confirmed by western blotting with the detection of a single protein band at ∼18 kDa. MANF was mainly present in neurons of the ITGC, consistent with the known expression pattern of MANF in the aging human cerebral cortex^1^ and adult mouse brain (Lindholm et al., 2008; Tseng et al., 2017; Yang et al., 2017; Danilova et al., 2019). This was also confirmed by MANF/NeuN double immunolabeling experiments.

The size of nucleoli of ITGC neurons was significantly smaller in pre-AD and AD than in control brains, in line with previous observations (Hu et al., 2003); however, MANF immunoreactivity in neurons was higher in patients than in controls. GRP78 and MANF interact as part of a macromolecular complex in the ER (Glembotski et al., 2012). AD is related to ER calcium deficiency (Trychta et al., 2018); depletion of ER calcium leads to dissociation of the MANF/GRP78 complex and MANF secretion (Apostolou et al., 2008; Glembotski et al., 2012). ER calcium depletion also causes ER stress, which further results in the upregulation of MANF. In the present study, double immunolabeling of MANF/GRP78 showed that the MANF seems localized within ER. Based on these findings, we speculate that in pre-AD and AD, calcium depletion and severe chronic ER stress leads to the upregulation of MANF and activation of the apoptosis signaling pathway, resulting in the shrinkage of neuronal nucleoli and neuronal death.

A large number of neurons in the ITGC express both MANF and NeuN, indicating that MANF is a protein expressed primarily in neurons. MANF expressed by adeno-associated virus (AAV) was predominantly detected in the cytoplasm of infected cells (Yang et al., 2017), which is consistent with the localization of endogenous MANF. AAV-induced MANF expression was also observed in neurons and glia of the cerebral cortex following ischemia (Airavaara et al., 2010). MANF is upregulated in neurons under pathologic conditions such as focal cerebral ischemia, and a larger infarct area was observed in MANF-deficient brains (Shen et al., 2012), suggesting that MANF exerts a protective effect against ischemic injury in cortical neurons (Mätlik et al., 2018). However, a larger sample size and more detailed cytologic analyses are needed to confirm our results and to determine whether the increased level of MANF in the pre-AD and AD brain is related to a perturbation of ER homeostasis.

There were some limitations to our study, we did not examine the expression of ER stress or apoptosis markers to clarify the function of MANF in AD. Nonetheless, our results demonstrate that MANF is overexpressed in neurons in the brain of pre-AD and AD patients, suggesting that it can serve as a diagnostic marker for early stage disease.

## Data Availability Statement

The original contributions presented in the study are included in the article/supplementary material, further inquiries can be directed to the corresponding authors.

## Ethics Statement

Paraffin-embedded postmortem human brain specimens were obtained via the Netherlands Brain Bank with informed written consent from the patients or their next of kin for the autopsy and use of brain material and clinical files for research purposes. The patients/participants provided their written informed consent to participate in this study.

## Author Contributions

X-CL designed the study, performed the immunohistochemistry and statistical analysis, and drafted the manuscript. X-HQ conducted the tissue preparation and parts of the experiments. HF and K-QZ participated in the study design and the statistical analysis. G-HC and Q-SW conceived, designed, and supervised all aspects of the study and revised the manuscript. All authors read and approved the final manuscript.

## Conflict of Interest

The authors declare that the research was conducted in the absence of any commercial or financial relationships that could be construed as a potential conflict of interest.

## Footnotes

1 https://www.proteinatlas.org/ENSG00000145050-MANF/tissue

## References

[B1] Adaikkan C. Middleton S. J. Marco A. Pao P. C. Mathys H. Kim D. N. (2019). Gamma entrainment binds higher-order brain regions and offers neuroprotection. *Neuron* 102 929–943. e8. 10.1016/j.neuron.2019.04.011 31076275 PMC6697125

[B2] Airavaara M. Chiocco M. J. Howard D. B. Zuchowski K. L. Peränen J. Liu C. (2010). Widespread cortical expression of MANF by AAV serotype 7: localization and protection against ischemic brain injury. *Exp. Neurol.* 225 104–113. 10.1016/j.expneurol.2010.05.020 20685313 PMC2925275

[B3] Alzheimer’s Association (2020). Alzheimer’s disease facts and figures. *Alzheimers Dement.* 16 391–460. 10.1002/alz.12068 32157811

[B4] Apostolou A. Shen Y. X. Liang Y. Luo J. Fang S. Y. (2008). Armet, a UPR-upregulated protein, inhibits cell proliferation and ER stress-induced cell death. *Exp. Cell Res.* 314 2454–2467. 10.1016/j.yexcr.2008.05.001 18561914 PMC6719340

[B5] Arrieta A. Blackwood E. A. Stauffer W. T. Santo Domingo M. Bilal A. S. Thuerauf D. J. (2020). Mesencephalic astrocyte-derived neurotrophic factor is an ER-resident chaperone that protects against reductive stress in the heart. *J. Biol. Chem.* 295 7566–7583. 10.1074/jbc.ra120.013345 32327487 PMC7261788

[B6] Braak H. Braak E. (1991). Neuropathological stageing of Alzheimer-related changes. *Acta Neuropathol.* 82 239–259. 10.1007/BF00308809 1759558

[B7] Braak H. Del Tredici K. (2018). Spreading of tau pathology in sporadic Alzheimer’s disease along cortico-cortical top-down connections. *Cereb. Cortex* 28 3372–3384. 10.1093/cercor/bhy152 29982389 PMC6095209

[B8] Bravo R. Parra V. Gatica D. Rodriguez A. E. Torrealba N. Paredes F. (2013). Endoplasmic reticulum and the unfolded protein response: dynamics and metabolic integration. *Int. Rev. Cell Mol. Biol.* 301 215–290. 10.1016/B978-0-12-407704-1.00005-1 23317820 PMC3666557

[B9] Cabral-Miranda F. Hetz C. (2018). ER stress and neurodegenerative disease: a cause or effect relationship? *Curr. Top. Microbiol. Immunol.* 414 131–157. 10.1007/82_2017_52 28864830

[B10] Danilova T. Galli E. Pakarinen E. Palm E. Lindholm P. Saarma M. (2019). Mesencephalic astrocyte-derived neurotrophic factor (MANF) is highly expressed in mouse tissues with metabolic function. *Front. Endocrinol. (Lausanne).* 10:765. 10.3389/fendo.2019.00765 31781038 PMC6851024

[B11] Gao L. Xu W. Fan S. Li T. Zhao T. Ying G. (2018). MANF attenuates neuronal apoptosis and promotes behavioral recovery via Akt/MDM-2/p53 pathway after traumatic spinal cord injury in rats. *BioFactors (Oxford, England).* 44 369–386. 10.1002/biof.1433 29797541

[B12] Gerakis Y. Hetz C. (2018). Emerging roles of ER stress in the etiology and pathogenesis of Alzheimer’s disease. *FEBS J.* 285 995–1011. 10.1111/febs.14332 29148236

[B13] Ghemrawi R. Khair M. (2020). Endoplasmic reticulum stress and unfolded protein response in neurodegenerative diseases. *Int. J. Mol. Sci.* 21:6127. 10.3390/ijms21176127 32854418 PMC7503386

[B14] Glembotski C. C. Thuerauf D. J. Huang C. Vekich J. A. Gottlieb R. A. Doroudgar S. (2012). Mesencephalic astrocyte-derived neurotrophic factor protects the heart from ischemic damage and is selectively secreted upon sarco/endoplasmic reticulum calcium depletion. *J. Biol. Chem.* 287 25893–25904. 10.1074/jbc.M112.356345 22637475 PMC3406674

[B15] Goswami P. Afjal M. A. Akhter J. Mangla A. Khan J. Parvez S. (2020). Involvement of endoplasmic reticulum stress in amyloid β (1-42)-induced Alzheimer’s like neuropathological process in rat brain. *Brain Res. Bull.* 165 108–117. 10.1016/j.brainresbull.2020.09.022 33011197

[B16] Hao F. Yang C. Chen S. S. Wang Y. Y. Zhou W. Hao Q. (2017). Long-term protective effects of AAV9-mesencephalic astrocyte-derived neurotrophic factor gene transfer in parkinsonian rats. *Exp. Neurol.* 291 120–133. 10.1016/j.expneurol.2017.01.008 28131727

[B17] Hellman M. Arumäe U. Yu L. Y. Lindholm P. Peränen J. Saarma M. (2011). Mesencephalic astrocyte-derived neurotrophic factor (MANF) has a unique mechanism to rescue apoptotic neurons. *J. Biol. Chem.* 286 2675–2680. 10.1074/jbc.M110.146738 21047780 PMC3024763

[B18] Herranen A. Ikäheimo K. Lankinen T. Pakarinen E. Fritzsch B. Saarma M. (2020). Deficiency of the ER-stress-regulator MANF triggers progressive outer hair cell death and hearing loss. *Cell Death Dis.* 11:100. 10.1038/s41419-020-2286-6 32029702 PMC7005028

[B19] Hu X. Y. Qin S. Lu Y. P. Ravid R. Swaab D. F. Zhou J. N. (2003). Decreased estrogen receptor-alpha expression in hippocampal neurons in relation to hyperphosphorylated tau in Alzheimer patients. *Acta Neuropathol.* 106 213–220. 10.1007/s00401-003-0720-3 12819990

[B20] Hu X. Y. Zhang H. Y. Qin S. Xu H. Swaab D. F. Zhou J. N. (2002). Increased p75(NTR) expression in hippocampal neurons containing hyperphosphorylated tau in Alzheimer patients. *Exp. Neurol.* 178 104–111. 10.1006/exnr.2002.8018 12460612

[B21] Huang Z. H. Ni R. J. Luo P. H. Zhou J. N. (2020). Distribution of tyrosine-hydroxylase-immunoreactive neurons in the hypothalamus of tree shrews. *J. Comp. Neurol.* 528 935–952. 10.1002/cne.24803 31674014

[B22] Lacosta A. M. Insua D. Badi H. Pesini P. Sarasa M. (2017). Neurofibrillary tangles of Aβx-40 in Alzheimer’s disease brains. *J. Alzheimers Dis.* 58 661–667. 10.3233/JAD-170163 28453491

[B23] Lindahl M. Saarma M. Lindholm P. (2017). Unconventional neurotrophic factors CDNF and MANF: Structure, physiological functions and therapeutic potential. *Neurobiol. Dis.* 97 90–102. 10.1016/j.nbd.2016.07.009 27425895

[B24] Lindholm P. Peränen J. Andressoo J. O. Kalkkinen N. Kokaia Z. Lindvall O. (2008). MANF is widely expressed in mammalian tissues and differently regulated after ischemic and epileptic insults in rodent brain. *Mol. Cell. Neurosci.* 39 356–371. 10.1016/j.mcn.2008.07.016 18718866

[B25] Lindholm P. Saarma M. (2010). Novel CDNF/MANF family of neurotrophic factors. *Dev. Neurobiol.* 70 360–371. 10.1002/dneu.20760 20186704

[B26] Liu Y. G. Zhang J. X. Jiang M. Cai Q. Fang J. M. Jin L. J. (2018). MANF improves the MPP+/MPTP-induced Parkinson’s disease via improvement of mitochondrial function and inhibition of oxidative stress. *Am. J. Transl. Res.* 10 1284–1294. 29887945 PMC5992546

[B27] Lu J. M. Luo L. Y. Huang D. Q. Liu X. Xia X. Wang Z. Y. (2018). Photoreceptor protection by mesencephalic astrocyte-derived neurotrophic factor (MANF). *eNeuro* 5 ENEURO.109–ENEURO.118. 10.1523/ENEURO.0109-18.2018 29687079 PMC5909182

[B28] Lu M. Lawrence D. A. Marsters S. Acosta-Alvear D. Kimmig P. Mendez A. S. (2014). Opposing unfolded-protein-response signals converge on death receptor 5 to control apoptosis. *Science* 345 98–101. 10.1126/science.1254312 24994655 PMC4284148

[B29] Masters C. L. Bateman R. Blennow K. Rowe C. C. Sperling R. A. Cummings J. L. (2015). Alzheimer’s disease. *Nat. Rev. Dis. Primers* 1:15056. 10.1038/nrdp.2015.56 27188934

[B30] Mathys H. Davila-Velderrain J. Peng Z. Gao F. Mohammadi S. Young J. Z. (2019). Single-cell transcriptomic analysis of Alzheimer’s disease. *Nature* 570 332–337. 10.1038/s41586-019-1195-2 31042697 PMC6865822

[B31] Mätlik K. Anttila J. E. Kuan-Yin T. Smolander O. P. Pakarinen E. Lehtonen L. (2018). Poststroke delivery of MANF promotes functional recovery in rats. *Sci. Adv.* 4:eaa8957. 10.1126/sciadv.aap8957 29806020 PMC5966223

[B32] Mégevand P. Groppe D. M. Bickel S. Mercier M. R. Goldfinger M. S. Keller C. J. (2017). The hippocampus and amygdala are integrators of neocortical influence: a corticocortical evoked potential study. *Brain Connect* 7 648–660. 10.1089/brain.2017.0527 28978234 PMC5915225

[B33] Meunier M. Barbeau E. (2013). Recognition memory and the medial temporal lobe: From monkey research to human pathology. *Rev. Neurol. (l. (Paris).* 169 459–469. 10.1016/j.neurol.2013.01.623 23473622

[B34] Mukherjee A. Soto C. (2011). Role of calcineurin in neurodegeneration produced by misfolded proteins and endoplasmic reticulum stress. *Curr. Opin. Cell Biol.* 23 223–230. 10.1016/j.ceb.2010.12.006 21295458 PMC3078182

[B35] Neves J. Zhu J. Sousa-Victor P. Konjikusic M. Riley R. Chew S. (2016). Immune modulation by MANF promotes tissue repair and regenerative success in the retina. *Science* 353:aaf3646. 10.1126/scienceaaf3646 PMC5270511 27365452

[B36] Pakarinen E. Danilova T. Võikar V. Chmielarz P. Piepponen P. Airavaara M. (2020). MANF ablation causes prolonged activation of the UPR without neurodegeneration in the mouse midbrain dopamine system. *eNeuro* 7 ENEURO.477–ENEURO.419. 10.1523/ENEURO.0477-19.2019 32005751 PMC7053174

[B37] Petrova P. Raibekas A. Pevsner J. Vigo N. Anafi M. Moore M. K. (2003). MANF: a new mesencephalic, astrocyte-derived neurotrophic factor with selectivity for dopaminergic neurons. *J. Mol. Neurosci.* 20 173–188. 10.1385/jmn:20:2:173 12794311

[B38] Pietronigro E. Zenaro E. Constantin G. (2016). Imaging of leukocyte trafficking in Alzheimer’s disease. *Front. Immunol.* 7:33. 10.3389/fimmu.2016.00033 26913031 PMC4753285

[B39] Rahman S. Archana A. Jan A. T. Minakshi R. (2018). Dissecting endoplasmic reticulum unfolded protein response (UPRER) in managing clandestine modus operandi of Alzheimer’s disease. *Front. Aging Neurosci.* 10:30. 10.3389/fnagi.2018.00030 29467648 PMC5808164

[B40] Sakono M. Kidani T. (2017). ATP-independent inhibition of amyloid beta fibrillation by the endoplasmic reticulum resident molecular chaperone GRP78. *Biochem. Biophys. Res. Commun.* 493 500–503. 10.1016/j.bbrc.2017.08.162 28870813

[B41] Scheff S. W. Price D. A. Schmitt F. A. Scheff M. A. Mufson E. J. (2011). Synaptic loss in the inferior temporal gyrus in mild cognitive impairment and Alzheimer’s disease. *J. Alzheimers Dis.* 24 547–557. 10.3233/JAD-2011-101782 21297265 PMC3098316

[B42] Scheltens P. Blennow K. Breteler M. M. de Strooper B. Frisoni G. B. Salloway S. (2016). Alzheimer’s disease. *Lancet* 388 505–517. 10.1016/S0140-6736(15)01124-1 26921134

[B43] Schneider C. A. Rasband W. S. Eliceiri K. W. (2012). NIH Image to ImageJ: 25 Years of image analysis. *Nat. Methods.* 9 671–675. 10.1038/nmeth.2089 22930834 PMC5554542

[B44] Shen Y. J. Sun A. Wang Y. H. Cha D. Q. Wang H. P. Wang F. C. (2012). Upregulation of mesencephalic astrocyte-derived neurotrophic factor in glial cells is associated with ischemia-induced glial activation. *J. Neuroinflammation.* 9:254. 10.1186/1742-2094-9-254 23173607 PMC3576245

[B45] Sousa-Victor P. Neves J. Cedron-Craft W. Ventura P. B. Liao C. Y. Riley R. R. (2019). MANF regulates metabolic and immune homeostasis in ageing and protects against liver damage. *Nat. Metab.* 1 276–290. 10.1038/s42255-018-0023-6 31489403 PMC6727652

[B46] Thangavel R. Van Hoesen G. W. Zaheer A. (2008). Posterior parahippocampal gyrus pathology in Alzheimer’s disease. *Neuroscience* 154 667–676. 10.1016/j.neuroscience.2008.03.077 18486350 PMC2517248

[B47] Trychta K. A. Bäck S. Henderson M. J. Harvey B. K. (2018). KDEL receptors are differentially regulated to maintain the ER proteome under calcium deficiency. *Cell Rep.* 25 1829.–1840. 10.1016/j.celrep.2018.10.055 1829–1840.e6, 30428351 PMC7336508

[B48] Tseng K. Y. Danilova T. Domanskyi A. Saarma M. Lindahl M. Airavaara M. (2017). MANF is essential for neurite extension and neuronal migration in the developing cortex. *eNeuro* 4 ENEURO.214–ENEURO.217. 10.1523/ENEURO.0214-17.2017 29082311 PMC5655607

[B49] Voutilainen M. H. Bäck S. Pörsti E. Toppinen L. Lindgren L. Lindholm P. (2009). Mesencephalic astrocyte-derived neurotrophic factor is neurorestorative in rat model of Parkinson’s disease. *J. Neurosci.* 29 9651–9659. 10.1523/JNEUROSCI.0833-09.2009 19641128 PMC6666534

[B50] Wang H. P. Ke Z. J. Alimov A. Xu M. Frank J. A. Fang S. Y. (2014). Spatiotemporal expression of MANF in the developing rat brain. *PLoS One.* 9:e90433. 10.1371/journal.pone.0090433 24587361 PMC3938758

[B51] Wang P. Yang Y. Pang G. Zhang C. Wei C. Tao X. (2021). Hepatocyte-derived MANF is protective for rifampicin-induced cholestatic hepatic injury via inhibiting ATF4-CHOP signal activation. *Free Radic Biol Med* 162 283–297. 10.1016/j.freeradbiomed.2020.10.028 33127565

[B52] Wang Y. Wen W. Li H. Clementino M. Xu H. Xu M. (2021). MANF is neuroprotective against ethanol-induced neurodegeneration through ameliorating ER stress. *Neurobiol. Dis.* 148:105216. 10.1016/j.nbd.2020.105216 33296727 PMC7856049

[B53] Xu S. C. Di Z. M. He Y. F. Wang R. J. Ma Y. Y. Sun R. (2019). Mesencephalic astrocyte-derived neurotrophic factor (MANF) protects against Aβ toxicity via attenuating Aβ-induced endoplasmic reticulum stress. *J. Neuroinflammation.* 16:35. 10.1186/s12974-019-1429-0 30760285 PMC6373169

[B54] Yan Y. Rato C. Rohland L. Preissler S. Ron D. (2019). MANF antagonizes nucleotide exchange by the endoplasmic reticulum chaperone BiP. *Nat. Commun.* 10:541. 10.1038/s41467-019-08450-4 30710085 PMC6358605

[B55] Yang S. Huang S. S. Gaertig M. A. Li X. J. Li S. H. (2014). Age-dependent decrease in chaperone activity impairs MANF expression, leading to Purkinje cell degeneration in inducible SCA17 mice. *Neuron* 81 349–365. 10.1016/j.neuron.2013.12.002 24462098 PMC4863472

[B56] Yang S. Yang H. M. Chang R. B. Yin P. Yang Y. Yang W. L. (2017). MANF regulates hypothalamic control of food intake and body weight. *Nat. Commun.* 8:579. 10.1038/s41467-017-00750-x 28924165 PMC5603516

[B57] Yang Y. Wang P. Zhang C. Huang F. Pang G. Wei C. (2021). Hepatocyte-derived MANF alleviates hepatic ischaemia-reperfusion injury via regulating endoplasmic reticulum stress-induced apoptosis in mice. *Liver Int.* 41 623–639. 10.1111/liv.14697 33064897

[B58] Zhu H. Y. Guo H. F. Hou H. L. Liu Y. J. Sheng S. L. Zhou J. N. (2007). Increased expression of the Nogo receptor in the hippocampus and its relation to the neuropathology in Alzheimer’s disease. *Hum. Pathol.* 38 426–434. 10.1016/j.humpath.2006.09.010 17188332

[B59] Zhu L. N. Chen D. Xu D. Chen S. H. Wang H. J. Liu L. (2017). Mesencephalic astrocyte-derived neurotrophic factor and its role in nervous system disease. *Neurol. Sci.* 38 1741–1746. 10.1007/s10072-017-3042-2 28685189

